# Comparative Analysis of Genetic Structure and Diversity in *Salvelinus fontinalis*, *Salvelinus malma*, and Their Hybrids Based on Microsatellite Loci

**DOI:** 10.3390/biology15151269

**Published:** 2026-08-03

**Authors:** Yuting Liu, Chunmei Yan, Haibo Li, Jiasong Zhang, Xiulan Wang, Tianyang Sun, Zhiguo Xiao

**Affiliations:** 1 Jilin Fisheries Research Institute, Changchun 130033, China; 18621093036@163.com (Y.L.);; 2 Key Laboratory of Freshwater Aquatic Genetic Resources, Ministry of Agriculture and Rural Affairs, Shanghai Ocean University, Shanghai 201306, China

**Keywords:** fish, hybridization, *Salvelinus*, genetic polymorphism, microsatellite genotype

## Abstract

This study compared the genetic diversity and structure of *Salvelinus fontinalis* (SF), *Salvelinus malma* (SM), and their hybrids (♂ SM × ♀ SF, FM) using 10 microsatellite loci. Our results showed that the three groups of populations had moderate-to-high diversity, with an average polymorphism information content (PIC) ranging from 0.4534 to 0.5400, and the genetic diversity was the highest in FM. This indicates that FM is a suitable breeding material and has the potential to enhance germplasm resources. Based on STRUCTURE analysis (optimal K = 3), we observed that the hybrid population was between the two parental populations, and UPGMA clustering showed that the hybrid population had a closer relationship with the maternal parent. This study can provide a theoretical basis and strategic reference for the efficient development, conservation and utilization of new hybrid *Salvelinus* germplasm resources; the hybrid *Salvelinus* can serve as an excellent breeding material and has important application value in the germplasm genetic improvement and variety selection of *S. fontinalis* and *S. malma*.

## Simple Summary

Interspecific hybridization serves as a feasible approach to enrich genetic diversity and explore novel germplasm resources in aquatic breeding. *Salvelinus malma* and *Salvelinus fontinalis* exhibit complementary breeding traits, whereas the genetic characteristics of their hybrid progeny remain largely unclear. In this study, ten microsatellite markers were applied to comparatively analyze the genetic diversity, population structure, and genetic relationships among *S. malma* (SM), *S. fontinalis* (SF), and their interspecific hybrid progeny (FM). The results suggested that the FM hybrid population showed relatively higher genetic diversity compared with the SF and SM parental populations. Hybrid individuals displayed intermediate genetic characteristics between the two parental species and tended to be genetically closer to the maternal SF population. Moderate genetic differentiation was observed across the three groups, with most genetic variation occurring at the individual level. The artificially produced hybrids harbored admixed genetic components inherited from both parents, implying potential value for germplasm improvement. Meanwhile, this study highlights possible ecological risks arising from hybrid escape and subsequent genetic introgression in wild charr populations. The results may provide preliminary genetic data and theoretical references for the utilization, genetic improvement, and conservation of cold-water *Salvelinus* germplasm resources.

## 1. Introduction

In this study, two economically important charr species, *Salvelinus malma* and *Salvelinus fontinalis*, were selected for interspecific hybridization analysis based on their distinctly complementary biological and aquacultural traits. *Salvelinus malma* is a native cold-water fish with excellent cold tolerance and superior flesh quality, conferring high market value; however, its slow growth rate severely limits aquaculture productivity and large-scale promotion. In contrast, *Salvelinus fontinalis* exhibits fast growth and strong disease resistance, which greatly improves farming efficiency, but its relatively low commercial price restricts its economic benefits. Interspecific hybridization between these two species provides a promising breeding strategy to integrate their respective advantageous traits and overcome the intrinsic industrial bottlenecks of both parental species. Despite the potential breeding value of this hybrid combination, systematic investigations on the genetic diversity and population structure of their hybrid progeny remain absent. At present, relevant research reports on *S. fontinalis* and *S. malma* are relatively scarce, and existing studies mainly focus on the fields of population genetic differentiation and phylogenetic evolution of the two species [1,2]. Therefore, the present study fills an important research gap and [3] offers novel genetic insights into the germplasm improvement and selective breeding of cold-water *Salvelinus* fish.

High genetic diversity can effectively improve the growth rate and disease resistance of cultured populations, and hybridization is an effective strategy to enhance genetic diversity [4,5]. Hybridization refers to the process in which two parents with different genotypes or genetic backgrounds mate and produce offspring through genetic recombination [6]. According to the genetic distance between parents, hybridization is divided into close hybridization and interspecific hybridization. Hybridization between species with a large genetic distance is called interspecific hybridization. This hybridization method combines traits from different genera or families, and genetic recombination can produce new genetic variations and even new species [7,8,9]. The comprehensive performance of hybrid individuals with variations may exceed that of their parents [10,11]. For example, the reciprocal hybrids of *Larimichthys polyactis* and *Larimichthys crocea* exhibit significant growth advantages and higher nutritional value [12,13]. The F_1_ offspring obtained by hybridization between *Micropterus salmoides salmoides* and *Micropterus salmoides floridanus* have significantly improved survival rate and growth rate [14]. The hybrid offspring of *Tachysurus fulvidraco* ♀ × *Pseudobagrus vachellii* ♂ have stronger immune adaptability [15]. Similarly, we initiated interspecific hybridization with *Salvelinus malma* as the male parent and *Salvelinus fontinalis* as the female parent in 2020, and successfully cultivated hybrid offspring with a fast growth rate and high nutritional value.

Microsatellite markers are defined by three characteristics: codominant inheritance, high mutation rates, and locus-specific variations. The capability to discern subtle population differentiation with a high degree of precision has led to the extensive utilization of these methods in the evaluation of genetic structure and diversity in a multitude of fish species [16,17,18,19,20]. In recent years, microsatellite markers have been successfully developed and applied to assess the genetic diversity of *Oncorhynchus mykiss* [21], *Salvelinus leucomaenis* [22], and *Salvelinus fontinalis* [23,24]. However, no published research has been found on the genetic diversity analysis of Salvelinus species and their interspecific hybrid offspring. To further explore the effects of crossbreeding on fish genetic diversity and clarify the genetic relationships between hybrid offspring and their parental populations, 10 microsatellite loci were used to conduct a comprehensive genetic diversity analysis, aiming to elucidate the genetic relationships and population differentiation between hybrid progeny and parental populations. This study provides a scientific theoretical basis for future crossbreeding and germplasm improvement of *Salvelinus* species.

## 2. Materials and Methods

### 2.1. Experimental Site and Sample Collection

*Salvelinus fontinalis* was introduced from Canada in 2001, and an artificial self-propagation system was established at Fushunxiang Cold-water Fish Co., Ltd. (Erdaobaihe Town, Antu County, Yanbian Korean Autonomous Prefecture, Jilin Province, China), Changbai Mountain Protection and Development Zone. All female broodstock of *Salvelinus fontinalis* used in this experiment were selected from the self-bred population of this base and were raised to the age of 3^+^; *Salvelinus malma* was collected from wild individuals in the Changbai River section of the upper reaches of the Yalu River. After artificial domestication and breeding in this fish farm, they were used as the experimental male broodstock (3^+^ years old).

**Figure 1:**
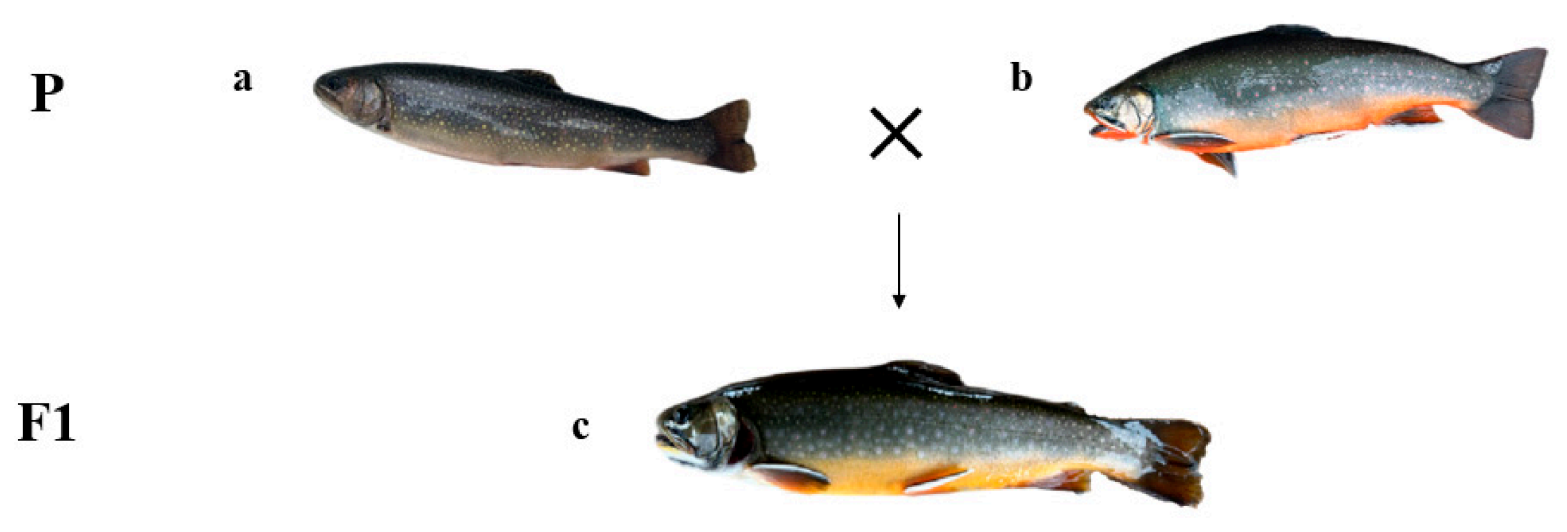
Diagram of the three populations. P—the parental generation; F_1_—the hybrid offspring; (**a**) *Salvelinus fontinalis*, SF; (**b**) *Salvelinus malma*, SM; (**c**) F_1_ hybrid progeny from ♀ *S. fontinalis* × ♂ *S. malma*, FM.

In this study, we performed interspecific hybridization with *Salvelinus fontinalis* serving as the female parent and *Salvelinus malma* as the male parent (♀ *S. fontinalis* × ♂ *S. malma*). Six mating groups were established at a female-to-male ratio of 6:1. The resulting first filial generation hybrids were designated FM (FM = F_1_ hybrid progeny from ♀ *S. fontinalis* × ♂ *S. malma*) (Figure 1). Morphological divergence was clearly observed among the three groups. *S. malma* possessed a pale yellowish-green body with sparse faint spots, while *S. fontinalis* showed dark gray-brown skin covered with dense distinct white spots. Their F_1_ hybrid displayed intermediate phenotypic characteristics: dark body background and dense pale spots inherited from maternal SF, accompanied by striking orange-yellow pelvic fins derived from paternal SM, representing a typical admixed morphological pattern of interspecific hybrid offspring. All experimental fish were collected from Fushunxiang Cold-water Fish Co., Ltd., Changbai Mountain Protection and Development Zone. During the breeding period, the water temperature was maintained at 6.8–7.2 °C, pH at 6.7–7.5, and dissolved oxygen at 8–10 mg/L. The experiment was carried out in November, which corresponds to the natural breeding season of charr species.

A total of 96 individuals were collected, including parent individuals of *Salvelinus fontinalis* (SF, N = 34) with an average body weight of 450–500 g and parent individuals of *Salvelinus malma* (SM, N = 30) with an average body weight of 340–390 g and 32 individuals of F_1_ hybrid progeny from ♀ *S. fontinalis* × ♂ *S. malma* (FM, N = 32). Fin ray tissues were excised and preserved in 95% ethanol.

Genomic DNA was extracted using the QIAGEN DNeasy Blood & Tissue Kit (Hilden, Germany) following the manufacturer’s instructions. The integrity of the extracted DNA was assessed using 1% agarose gel electrophoresis, while the concentration and purity of DNA were determined using a NanoDrop 2000 micro ultraviolet-visible spectrophotometer (Thermo Fisher Scientific, Wilmington, DE, USA). All DNA samples were uniformly diluted to 50 ng/µL and stored at −20 °C for subsequent use.

### 2.2. Screening and Analysis of Microsatellite Markers

Ten pairs of microsatellite primers were screened from published studies. Primers were synthesized and labeled with FAM fluorescence at the 5′ end by Beijing Dingguo Changsheng Biotechnology Co., Ltd. (Beijing, China). All loci were amplified separately on independent PCR plates (single-locus amplification), and each amplified single-locus product was directly subjected to fragment analysis on the sequencer. After preliminary experiments and optimization of reaction conditions in our laboratory, primers with stable amplification were selected for subsequent genotyping. Detailed primer sequences are shown in Table S1. The 5′ end of each forward primer was labeled with FAM fluorescent dye.

The total volume of the PCR reaction system was 25 μL, containing 1 μL genomic DNA template (50 ng), 0.5 μL each of forward and reverse primers (20 μmol/L), 0.5 μL dNTPs, 2.5 μL 10 × PCR buffer, 0.5 μL Taq DNA polymerase, and 19.5 μL sterile double-distilled water. Negative control groups containing sterile water instead of DNA template were set up in each PCR plate to detect exogenous DNA contamination. The amplification procedure was as follows: initial denaturation at 94 °C for 4 min, followed by 5 touchdown cycles consisting of denaturation at 94 °C for 45 s, annealing from 60 °C to 55 °C with a 1 °C decrement per cycle, and extension at 72 °C for 60 s. Subsequently, 40 regular amplification cycles were performed: 94 °C for 30 s, 52 °C for 30 s, and 72 °C for 30 s. A final extension was carried out at 72 °C for 7 min. PCR products were stored at 4 °C.

Diluted fluorescent PCR products were subjected to 1.0% agarose gel electrophoresis to verify amplification quality. Qualified products were analyzed by capillary electrophoresis using an ABI 3730xl Genetic Analyzer (Applied Biosystems, Foster City, CA, USA). Fragment sizes were calibrated against the LIZ 500 size standard in Gene Mapper 4.0 software, and consistent allele binning was conducted. All electrophoretic peak profiles were manually inspected to eliminate misjudgments caused by stutter peaks. Loci and individuals with irregular amplification or null alleles were excluded. Primers with three or more alleles were defined as polymorphic markers. For genotyping quality control, approximately 20% of all DNA samples were randomly selected for repeated PCR amplification and capillary electrophoresis to assess repeatability. Genotyping error rate was calculated by comparing genotype data from the two independent replicates; any inconsistent allelic calls were manually rechecked and corrected. Genotyping was performed on qualified samples, and allelic information and genotype data for each locus were recorded.

Based on the genotypes of the three groups at different loci, we used POPGENE 3.2 [25] to analyze the genotypes of different loci across populations to calculate the following parameters for each microsatellite locus: the number of alleles (Na), the effective number of alleles (Ne), the observed heterozygosity (Ho), the expected heterozygosity (He), Shannon’s information index (I), Nei’s standard genetic distance, the inbreeding coefficient (Fis), and pairwise genetic differentiation coefficient (FST, Weir and Cockerham’s θ), the gene flow (Nm) and Hardy–Weinberg equilibrium (HWE) *p*-values. Bonferroni correction was applied to adjust *p*-values for multiple HWE tests across all loci to avoid false positive results. We also constructed phylogenetic trees among populations based on Nei’s genetic distance. Polymorphic information content (PIC) was calculated using Cervus 3.0.7 [26]. Molecular variance analysis (AMOVA) was conducted using Arlequin version 3.5.2.2 [27]. The GenAlEx 6.5 Excel add-in [28] was employed for chi-square tests of Hardy–Weinberg equilibrium, as well as principal coordinates analysis (PCoA) based on pairwise genetic distances among populations and individuals. We used Structure Harvester web version (online tool, Earl, 2012) [29] to calculate the ΔK statistic based on the Evanno method and determine the optimal genetic cluster number K. Population genetic structure was inferred using STRUCTURE version 2.3.4 [30], which determined the optimal K value based on two criteria: Mean lnP(K) [31] and ΔK [32]. The number of assumed genetic clusters (K) ranged from 1 to 9. For each K value, 20 independent runs were conducted with a burn-in of 10,000 steps followed by 100,000 Markov Chain Monte Carlo (MCMC) iterations. The optimal K was determined using the ΔK method, and the corresponding bar plot illustrating population stratification was constructed.

Data processing was performed using SPSS 23.0 software, and experimental results were expressed as mean ± standard deviation.

## 3. Results

### 3.1. Population Genetic Diversity

Ten pairs of microsatellite primers were used to evaluate the genetic diversity parameters, including the number of observed alleles (Na), effective number of alleles (Ne), observed heterozygosity (Ho), expected heterozygosity (He), Shannon’s information index (I), Nei’s standard genetic distance, and polymorphism information content (PIC) in SM, SF and FM (Table S2), while population-level summarized genetic diversity statistics and one-way ANOVA results for inter-population comparisons are presented in Table 1.

A total of 174 observed alleles (Na) were detected across the three populations, with an overall average Na value of 5.80. The Na of each locus ranged from 1 to 15. The locus OMM1032 showed the highest Na value (15) in the FM population, whereas OMM1088 presented the lowest Na value (1) in the SM population. The overall average He across all loci and populations was 0.5610, Shannon’s information index (I) of individual locus ranged from 0.1461 to 2.3670, and Nei’s standard genetic distance varied from 0.0000 to 0.8978.

The average I values of the SF (1.2243) and FM (1.2751) populations were both greater than 1, indicating a high level of genetic diversity. By contrast, the average I value of the SM population was 0.9345, reflecting relatively low genetic diversity. The average polymorphism information content of the three populations was 0.5080, with an overall mean PIC value greater than 0.5, suggesting that most microsatellite loci exhibited high polymorphism [33]. Hardy–Weinberg equilibrium tests were conducted across 10 microsatellite loci in three populations, and 22 locus–population combinations were consistent with HWE (Table S2). One-way ANOVA was further applied to compare Ho, He and PIC among SM, SF, and FM, and no significant inter-population differences were detected (*p* > 0.05 for all three indices, Table 1).

### 3.2. Genetic Structure

Based on 10 microsatellite markers, pairwise Fst and gene flow (Nm) between pairwise comparisons of the three populations were analyzed (Table 2). The values of Nm ranged from 0.7714 to 3.3809. The Nm between the FM and SF populations was the highest at 3.3809, while that between the SM and SF populations was the lowest at 0.7714. The maximum pairwise Fst value of 0.2448 was observed between the SM and SF populations, and the minimum pairwise Fst value of 0.0689 was detected between the SF and FM populations.

The genetic similarity coefficient and genetic distance among the three populations ranged from 0.3125 to 0.7661 and from 0.2664 to 1.1630, respectively (Table 3). The SF and FM populations exhibited the highest genetic similarity coefficient (0.7661) and the lowest genetic distance (0.2664). In contrast, the SF and SM populations showed the minimum genetic similarity coefficient (0.3125) and the maximum genetic distance (1.1630).

The AMOVA results revealed that the percentage of variation among populations was 15.81%; among individuals within populations, it was 24.48%; within individuals, it was 59.71%. These findings indicate that the majority of genetic variation resides within individuals. The F*_IT_* value was 0.402, meaning that the observed heterozygote frequency was 40.2% lower than the expected value across all samples. The F*_IS_* value of 0.290 reflects significant inbreeding within populations. In addition, the F*_ST_* value of 0.158 indicates moderate genetic differentiation among the three groups. This differentiation mainly stems from the inherent interspecific genetic divergence between the two parental species, rather than gene flow among conspecific populations (Table 4).

### 3.3. Genetic Clustering Analysis

**Figure 2:**
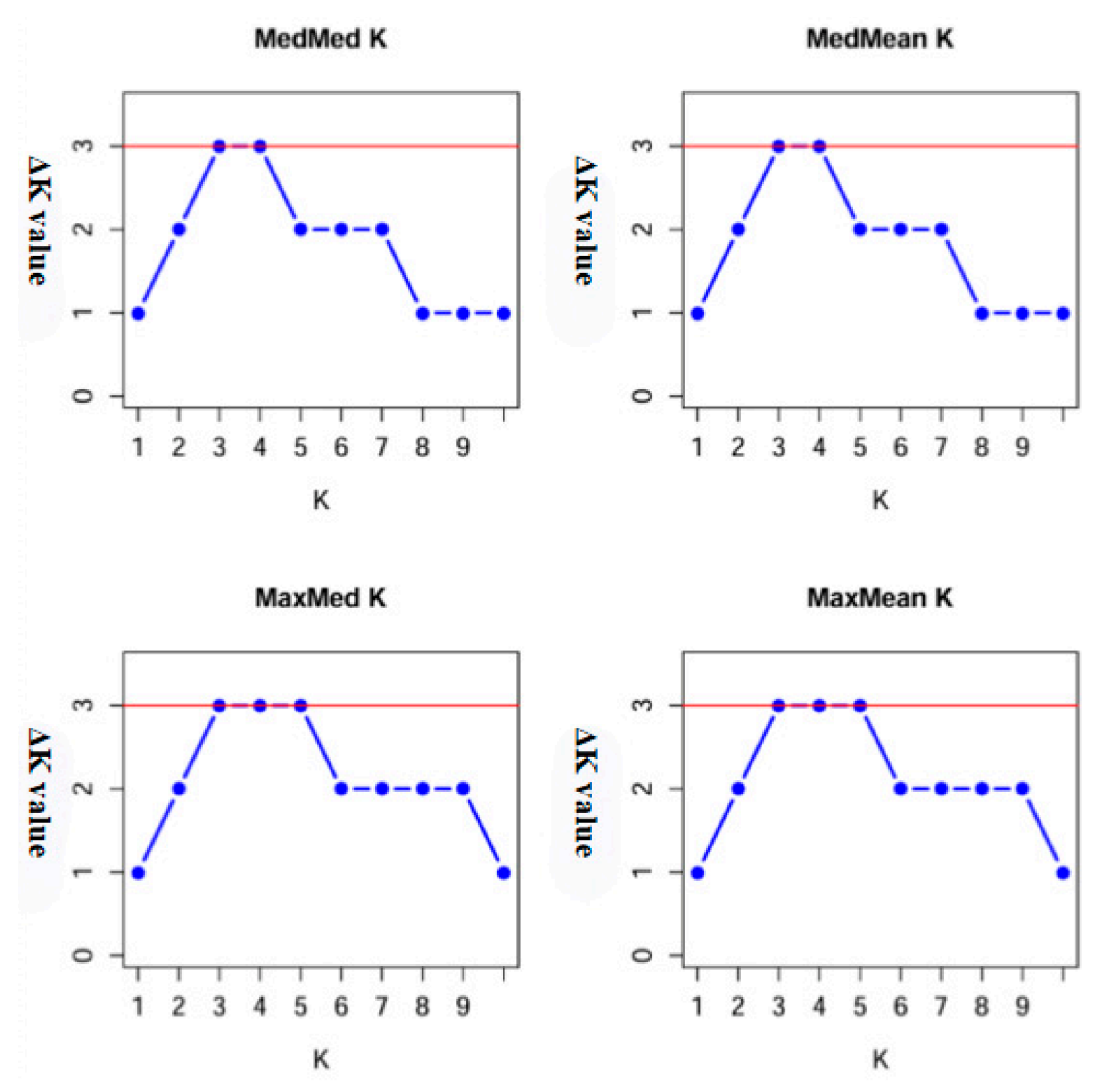
Evanno’s ΔK analysis to determine the optimal number of ancestral genetic clusters (K) across K = 1 to 9. Four alternative ΔK statistics (MedMed K, MedMean K, MaxMed K, MaxMean K) calculated by Structure Harvester for estimating the optimal number of ancestral genetic clusters (K) ranging from K = 1 to 9. The *x*-axis represents the predefined number of genetic clusters (K), and the *y*-axis denotes the corresponding ΔK value calculated by each algorithm. The red horizontal line highlights the peak ΔK value at K = 3, which was identified as the optimal number of ancestral genetic clusters across all four calculation methods.

**Figure 3:**
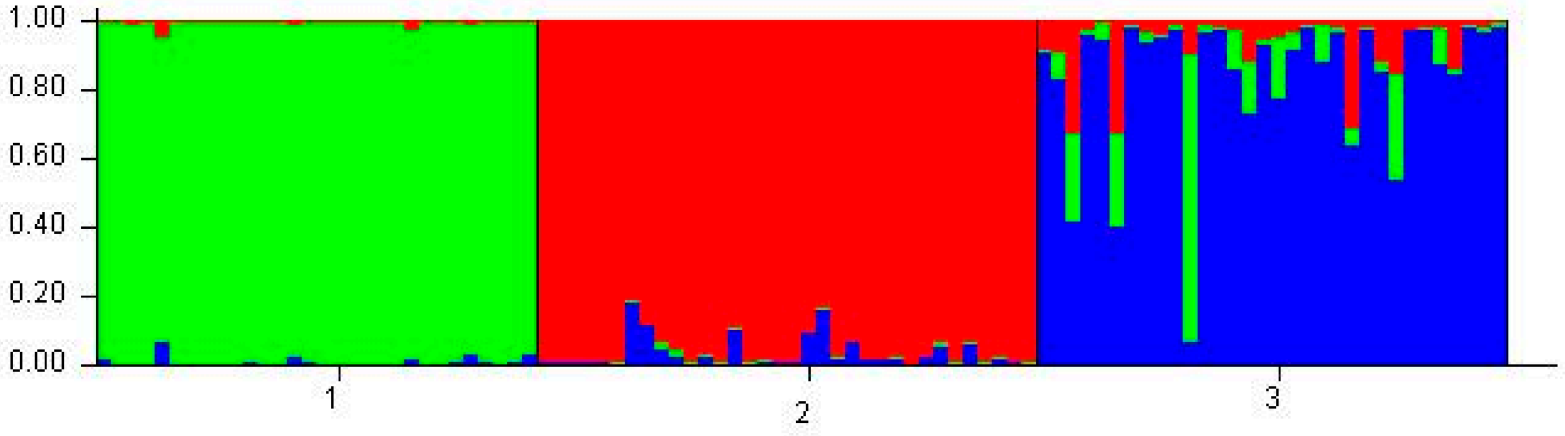
Genetic structure analysis of individuals from three populations under K = 3 hypothesis. Each vertical bar represents an individual, with different colors indicating the proportional contribution of each K genetic cluster to the individual’s genotype: 1. SM population, 2. SF population, 3. FM population.

**Figure 4:**
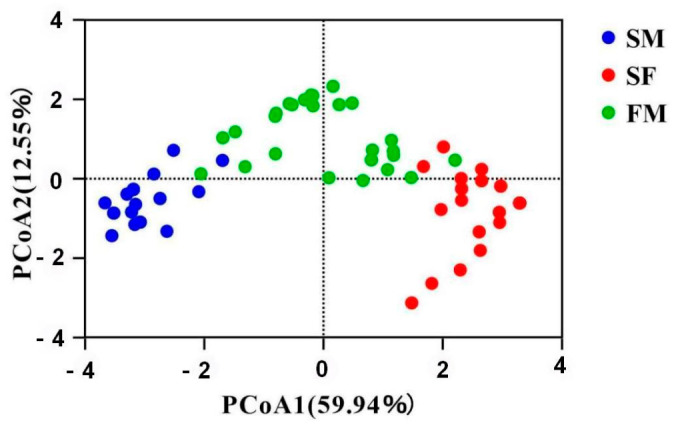
Principal coordinate analysis (PCoA) plot of individuals based on genetic distance. Group: SM = *Salvelinus malma*, SF = *Salvelinus fontinalis*, FM = *interspecific hybrid progeny*. PCoA1 and PCoA2 account for 59.94% and 12.55% of total genetic variance separately. Each point corresponds to one individual.

Figure 2, generated by Structure Harvester, illustrates the trend of ΔK variation, with the maximum ΔK peak detected at K = 3 according to the Evanno method. Figure 3, constructed based on the membership probability Q values of the 96 individuals, presents the clustering pattern at K = 3. However, considering all hybrid individuals in this study are artificial F_1_ progeny derived from two pure parental species, K = 2 is more biologically reasonable. The STRUCTURE barplot corresponding to K = 2 is provided in Supplementary Figure S1, where all F_1_ hybrids exhibit nearly equal genetic admixture from the two parental lineages, consistent with the genetic characteristics of first-generation interspecific hybrids. This clustering pattern was further validated by the results of principal coordinate analysis (PCoA) (Figure 4).

## 4. Discussion

### 4.1. Genetic Diversity

The level of genetic diversity is a key determinant of species growth performance, disease resistance, and environmental adaptability. Molecular markers can efficiently and accurately characterize the genetic features of populations. PIC, Na, Ne, Ho, He, and I are the core quantitative indices for evaluating the level of genetic diversity revealed by molecular markers. In general, higher values of the above indicators correspond to richer genetic diversity and greater genetic variation within populations. Specifically, a higher PIC value indicates more prominent polymorphism of the molecular marker in the target population, as well as stronger capacity to characterize population genetic diversity. In this study, ten microsatellite markers were used to analyze 96 fish individuals from three populations. The average PIC values of each population ranged from 0.4534 to 0.5400, indicating moderate-to-high polymorphism.

The average observed heterozygosity (*Ho*) across the three populations ranged from 0.4867 to 0.7471, while the average expected heterozygosity (*He*) varied between 0.4388 and 0.6286. These results indicated that the three populations in this study possessed a relatively high level of genetic diversity. Furthermore, we observed that the *Ho* was higher than the *He* at most loci, resulting in a negative inbreeding coefficient (*Fis* < 0). This suggests that outbreeding occurred at most loci in the studied populations, with no obvious inbreeding depression observed, which is conducive to improving the heterozygosity and genetic diversity of the populations [34]. The level of genetic diversity among the three populations could be ranked as FM > SF > SM. The hybrid FM population exhibited the highest genetic diversity, exceeding that of the two parental populations. This result is consistent with previous studies on *Larimichthys polyactis* ♀ × *Larimichthys crocea* ♂, *Larimichthys polyactis* ♂ × *Larimichthys crocea* ♀ [35], *Epinephelus fuscoguttatus* ♀ × *Epinephelus polyphekadion* ♂ [36], and *Epinephelus fuscoguttatus* ♀ × *Epinephelus tukula* ♂ [37]. It may be attributed to genetic recombination during hybridization breeding. Genetic recombination and segregation occur between parental genomes, generating abundant variant genotypes and thereby enhancing the genetic diversity of the species [6,11], and the characteristics of microsatellite markers adopted in this study help interpret the underlying allelic variation pattern more comprehensively. Microsatellites are widely recognized as co-dominant nuclear markers with relatively high mutation rates compared with other nuclear loci, which enables them to simultaneously capture allelic variants inherited from both maternal and paternal genomes rather than only detecting single parental alleles [38,39]. After interspecific crossing, divergent allele pools from SM and SF converge within hybrid individuals, generating apparent allele complementation at most genotyped loci; a set of private alleles unique to each parent can coexist in FM hybrids and expand the total allelic repertoire of the hybrid group [40,41]. In addition to the stacking of complementary parental alleles, meiotic recombination during gametogenesis may rearrange chromosomal fragments derived from two parent species, which could potentially produce novel allelic combinations that are rarely observed in pure parental populations [42,43]. The high mutability of microsatellite repeats further facilitates the accumulation of minor allelic variants over successive hybrid generations, which may further lift the overall polymorphism level of FM to a slight extent. It should be noted that such elevated genetic diversity in FM does not represent universal heterosis across all hybrid combinations, and the magnitude of diversity promotion may depend on the genetic divergence between parental species, crossing generations, and the effective population size of the hybrid progeny [44,45]. Consistent with our observations, similar patterns of moderately enhanced genetic diversity in interspecific char hybrids have been reported in previous salmonid research, where combined parental allelic resources and recombinant variants jointly contributed to higher polymorphism relative to single parental taxa [43,46].

We assessed the Hardy–Weinberg equilibrium (HWE) across the three populations using ten microsatellite markers, and varying degrees of disequilibrium were detected within each group. Locus OMM1032 showed extremely significant departures from HWE in two populations, demonstrating that the genotypic distribution at this locus deviated from the theoretical values expected under random mating. Locus-wise inbreeding coefficients further provided supporting evidence for the pattern of genotypic variation. Negative Fis values prevailed at most loci in SF and FM, a signature of heterozygote excess, which excludes consanguineous mating as a leading contributor to the observed disequilibrium. Multiple co-occurring factors could account for the disrupted HWE pattern: limited sampling scale, intrinsic mutational properties unique to certain microsatellite loci, undetected null alleles, divergent selective pressure acting on repeat regions, or subtle cryptic population subdivision that generates the Wahlund effect [26].

### 4.2. Genetic Structure

The results of this study reveal that the variation rate among populations is 15.81%, representing a small proportion, whereas the variation within individuals accounts for the largest share at 59.71%. This finding is consistent with previous reports on hybrid bass (*Ambloplites rupestris* and *Ambloplites cavifrons*) [47], indicating that inter-population genetic differentiation is the major contributor to overall genetic variation. The findings indicate a high level of allelic diversity within individuals and suggest limited differentiation among the three populations, likely due to frequent gene flow between them. Additionally, the FIT and FIS values for the four populations are significantly greater than zero (*p* < 0.001), indicating substantial inbreeding within the populations [3]. The value of *F_ST_* among the three populations indicated a middle level of genetic differentiation (*F_ST_* = 0.158) with statistically significant differences (*p* < 0.001). This further confirmed significant genetic divergence among populations and a low level of gene flow between them [3]. Comparison of genetic distance and genetic differentiation among the three populations revealed that FM and SF shared the closest genetic relationship, whereas the genetic divergence between SM and SF was the largest. This indicates that the hybrid progeny is genetically closer to the female parent. Phylogenetic tree analysis showed that the three populations could be divided into two distinct clusters, with the hybrid population clustering together with its female parent. This result is consistent with previous findings in hybrids of *Ambloplites rupestris* and *Ambloplites cavifrons* [47] and *Larimichthys crocea* ♀ × *Miichthys miiuy* ♂ [48]. Notably, although population differentiation analysis based on genetic distance can clearly reveal the overall differentiation pattern among populations, it has certain limitations in resolving the complex genetic structure of hybrid-origin populations. Previous studies have indicated that sample size, marker polymorphism, genetic distance algorithms, and clustering methods can all affect the reliability of genetic differentiation analysis [49]; meanwhile, hybridization events and population bottleneck events can interfere with genetic differentiation signals, thereby increasing the difficulty of resolving the genetic relationships among populations [50].

Notably, the results of principal coordinate analysis (PCoA) revealed that hybrid individuals were genetically intermediate between the two parental populations. This pattern was consistent with the genetic structure analysis but inconsistent with the UPGMA phylogenetic tree. Such discrepancies mainly arise from differences in algorithmic principles between the two methods. Phylogenetic cluster analysis prioritizes the reconstruction of evolutionary relationships and is strongly affected by allelic frequency variation. If certain alleles are enriched in one parental group, hybrid progeny will appear genetically closer to this parent in distance-based clustering. By contrast, PCoA evaluates comprehensive genetic relatedness across all loci in a multidimensional space. When alleles from both parents are widely shared across microsatellite loci, the overall genomic differentiation becomes weakened, and hybrid individuals display an admixed, intermediate genetic pattern between the two parents.

Beyond the intragenomic genetic characteristics of hybrid progeny, the occurrence of artificially produced interspecific hybrids in cultured charr populations also implies profound biological and ecological implications for germplasm conservation and aquatic ecosystem stability. In the present study, the FM hybrids exhibited elevated genetic diversity and admixed nuclear genetic components derived from both parental species, demonstrating that interspecific hybridization effectively reshapes genetic variation and enriches the genetic resource base of cultured Salvelinus populations. However, the existence of fertile hybrid individuals also raises potential ecological risks associated with artificial hybridization. If escaped hybrid individuals enter natural water bodies, gene introgression may occur between cultured hybrids and wild native charr populations, leading to genetic pollution, erosion of unique wild gene pools, and irreversible disruption of the long-term genetic integrity of indigenous Salvelinus germplasm resources [51,52]. From an ecological perspective, hybrid individuals with intermediate genetic backgrounds and potentially altered adaptive traits may compete ecologically with wild conspecifics or congeners, changing natural population structures, disturbing niche partitioning, and further altering the dynamic balance of local freshwater ecosystems [35]. Therefore, although artificial interspecific hybridization provides valuable germplasm materials for genetic improvement and selective breeding of cold-water fish, strict aquaculture isolation management and escape prevention measures are essential to avoid uncontrolled genetic introgression and ecological disturbance. This study highlights the necessity of balancing germplasm innovation utilization and wild genetic resource protection during artificial crossbreeding practice.

## 5. Conclusions

In this study, ten microsatellite loci were used to investigate the genetic diversity and population structure of *Salvelinus malma*, *Salvelinus fontinalis*, and their hybrids. The results showed that the genetic diversity ranked as FM > SF > SM, demonstrating that interspecific hybridization reshapes the genetic variation level of the hybrid offspring. The hybrid population harbors admixed genetic variants inherited from both parental char species and forms a distinct composite genetic background different from pure parental groups. This genetically diverse hybrid group can be used as ideal experimental material for further exploring interspecific genetic recombination and differentiation within the genus *Salvelinus*. Meanwhile, the present study also provides baseline genetic data to clarify the genetic differentiation pattern between the two parental species and their hybrid progeny.

We also highlight potential ecological risks brought by fertile hybrid progeny. If hybrid fish escape into natural water bodies, gene exchange between cultured hybrids and wild char populations will occur, resulting in irreversible genetic mixing of wild germplasm resources. To eliminate such ecological threats, developing all-sterile hybrid strains should be prioritized in follow-up research work. In aquaculture practice, standardized enclosure management must be carried out to cut off escape channels of hybrid individuals, so as to effectively protect the natural genetic integrity of wild *Salvelinus* populations.

## Figures and Tables

**Table 1:** Genetic diversity parameters of 10 microsatellite loci in three populations.

Group	Na	Ne	Ho	He	I	PIC	*p*-Value (Ho/He/PIC)
SM	4.80 ± 4.10	3.24 ± 3.10	0.49 ± 0.39	0.44 ± 0.35	0.93 ± 0.86	0.45	0.0939/0.1695/0.6926
SF	5.90 ± 3.14	2.95 ± 1.09	0.75 ± 0.26	0.62 ± 0.19	1.22 ± 0.47	0.53	0.0939/0.1695/0.6926
FM	6.70 ± 4.11	2.95 ± 1.06	0.73 ± 0.15	0.63 ± 0.14	1.28 ± 0.51	0.54	0.0939/0.1695/0.6926
Mean	5.80 ± 3.78	3.05 ± 2.01	0.65 ± 0.31	0.56 ± 0.25	1.14 ± 0.64	0.51	—

One-way analysis of variance (ANOVA) was performed to test for significant differences in Ho, He, and PIC among the three populations, with the corresponding *p*-values presented in the last column.

**Table 2:** Gene flow (Nm), pairwise genetic differentiation coefficient (Fst), Nei’s genetic identity, and genetic distance among populations.

Population Pair	FST	Nm	Nei’s Genetic Identity	Genetic Distance
SM vs. SF	0.2448 ***	0.7714	0.3125	1.1630 ***
SM vs. FM	0.1070 ***	2.0874	0.7503	0.2873 **
SF vs. FM	0.0689 *	3.3809	0.7661	0.2664 **

Group: SM = *Salvelinus malma*, SF = *Salvelinus fontinalis*, FM = *interspecific hybrid progeny*; FST: pairwise genetic differentiation coefficient; Nm: gene flow between population pairs; Nei’s genetic identity and genetic distance were calculated according to Nei. Significance thresholds: * *p* < 0.05, ** *p* < 0.01, *** *p* < 0.001.

**Table 3:** Nei’s genetic identity (diagonal above) and genetic distance (diagonal below) among populations.

Populations	SM	SF	FM
SM		0.3125	0.7503
SF	1.1630 ***		0.7661
FM	0.2873 **	0.2664 **	

Group: SM = *Salvelinus malma*, SF = *Salvelinus fontinalis*, FM = *interspecific hybrid progeny*. ** *p* < 0.01, *** *p* < 0.001.

**Table 4:** AMOVA of the three populations based on 10 microsatellite loci.

Source of Variance	Degree of Freedom	Sum of Squares of Mean Deviation	Estimated Variance	Percentage of Total Variance (%)	Fixation Indices
Among populations	2	75.73	0.628	15.81	F*_ST_* = 0.158 ***
Among individuals	93	661.1	0.975	24.48	F*_IS_* = 0.290 ***
Within individuals	95	512	2.37	59.71	F*_IT_* = 0.402 ***
Total	190	1248.83	3.973	100	

F*_IS_*: inbreeding coefficient within individuals, F*_ST_*: fixation index between subpopulations, F*_IT_*: inbreeding coefficient within subpopulations. *** *p* < 0.001.

## Data Availability

The raw data supporting the conclusions of this article will be made available by the authors on request.

## Supplementary Materials

The following supporting information can be downloaded at: https://www.mdpi.com/article/10.3390/biology15151269/s1, Table S1: Information of 10 pairs of microsatellite primers; Table S2: Locus-by-locus genetic diversity parameters and Hardy–Weinberg equilibrium test results for 10 microsatellite loci across three populations; Figure S1: Genetic structure analysis of individuals from three populations under K = 2 hypothesis.
