# Supplementary material for: Comparative Analysis of Genetic Structure and Diversity in Salvelinus fontinalis, Salvelinus malma, and Their Hybrids Based on Microsatellite Loci
Source: Biology (Basel). 2026 Aug 3;15(15):1269. doi: 10.3390/biology15151269 (PMC13464715; doi:10.3390/biology15151269)
Supplement: Supplementary file 1 [file biology-15-01269-s001.zip › biology-4376373-supplementary.pdf]

Table S1. Information of 10 pairs of microsatellite primers.

Locus	Primer Sequence(5′-3′)	Tm/◦C	Allele size (bp)	Fluorescent Labeling
AF346680	F: CCAGTGCTACCGACACTGAA<br>R: GCTGTCCTTGGAGAGACTGG	58	120 ～ 253	FAM
OM1016	F: TGCCCCCATGAGTAAACATA<br>R: TCTCTCTCCTTTCCTCTCTCCA	58	161 ～ 251	FAM
AF346673	F: CTTCTGGGGACAGATGGAAA<br>R: CAGAGAACAGCAGGAGTCCA	62	201 ～ 587	FAM
OMM5000	F: AACAGAGCAGTGAGGGGACTGAGA<br>R: CAAGTGATGTTGGTGCGAGGG	62	246 ～ 318	FAM
OMM5017	F: TTGAGCCAAACATGCCTC<br>R: CACAGCATCTAGACAGTTCCC	62	197 ～ 234	FAM
OMM1125	F: GGAGATTGGGTGAGAGCTAAA<br>R: TTCTCATCCCATCTACCATCC	62	113 ～ 161	FAM
OMM1088	F: CTACAGGCCAACACTACAATC<br>R: CTATAAAGGGAATAGGCACCT	62	113 ～ 170	FAM
OMM1077	F: GGCTGACCAGAGAAAGACTAGTTC<br>R: TGTTACGGTGTCTGACATGC	62	225 ～ 262	FAM
OMM1032	F: GCGAGGAAGAGAAAGTAGTAG<br>R: CCCATCTTCTCTCTGATTATG	62	200 ～ 222	FAM
HLJZ056	F: CTCTGTCTCATCTCGCTT<br>R: TTCACTTGGTGTAATGGC	58	219 ～ 232	FAM

Supplementary Table S2 Locus-by-locus genetic diversity parameters and Hardy–Weinberg equilibrium test results for 10 microsatellite loci across three populations

Locus	Group	Na	Ne	Ho	He	Nei	I	PIC	HWE
	SM	4	1.3667	0.2667	0.2729	0.2683	0.5720	0.5300	1.0000
AF346680	SF	5	2.2578	0.7353	0.5654	0.5571	1.1147	0.5370	1.0000
	FM	4	2.1289	0.8125	0.5387	0.5303	0.8322	0.4197	0.5600
	SM	12	7.5630	0.9333	0.8825	0.8678	2.2004	0.8770	1.0000
OM1016	SF	9	3.7655	0.9706	0.7454	0.7344	1.6563	0.7070	1.0000
	FM	11	4.7850	0.8438	0.8036	0.7910	1.8752	0.7740	0.0460
	SM	2	2.0000	1.0000	0.5085	0.5000	0.6931	0.5000	0.0000
AF346673	SF	2	1.1918	0.1765	0.1633	0.1609	0.2984	0.1530	1.0000
	FM	3	1.8893	0.6875	0.4782	0.4707	0.7482	0.3856	0.4000
	SM	2	1.0689	0.0667	0.0655	0.0644	0.1461	0.0640	1.0000
OMM5000	SF	9	3.1456	0.9412	0.6923	0.6821	1.4970	0.6730	0.0610
	FM	6	2.5924	0.8438	0.6240	0.6143	1.2567	0.5430	1.0000
	SM	6	4.4665	0.7333	0.7893	0.7761	1.6223	0.7760	0.0000
OMM5017	SF	4	3.1758	0.9412	0.6953	0.6851	1.2324	0.6330	0.0510
	FM	8	3.4830	0.8750	0.7242	0.7129	1.5514	0.6260	1.0000
	SM	5	2.7481	0.6667	0.6469	0.6361	1.1739	0.6200	1.0000
OMM1125	SF	4	2.1689	0.5588	0.5470	0.5389	0.9950	0.4970	0.0000
	FM	3	1.9523	0.4062	0.4955	0.4878	0.7346	0.3831	1.0000
	SM	1	1.0000	0.0000	0.0000	0.0000	0.0000	0.0000	1.0000
OMM1088	SF	2	1.9983	0.9706	0.5070	0.4996	0.6927	0.1570	0.0000
	FM	2	1.6787	0.5625	0.4107	0.4043	0.5941	0.3225	0.3330
	SM	2	1.2195	0.2000	0.1831	0.1800	0.3251	0.1800	1.0000
OMM1077	SF	8	5.0813	0.9118	0.8152	0.8032	1.7959	0.6260	1.0000
	FM	9	3.3032	0.7188	0.7083	0.6973	1.6415	0.6020	1.0000
OMM1032	SM	12	9.7826	0.8667	0.9130	0.8978	2.3670	0.8680	0.0000
	SF	11	3.3266	0.5294	0.7098	0.6994	1.6349	0.6670	0.0000
	FM	15	4.1626	0.6875	0.7718	0.7598	1.9939	0.7010	0.1130
HLJZ056	SM	2	1.1421	0.1333	0.1266	0.1244	0.2449	0.1190	1.0000
	SF	5	3.3851	0.7353	0.7151	0.7046	1.3253	0.6570	1.0000
	FM	6	3.5617	0.8125	0.7307	0.7192	1.5233	0.6420	1.0000

Group: SM = Salvelinus malma, SF = Salvelinus fontinalis, FM = interspecific hybrid progeny; Na observed number of alleles, Ne expected number of alleles, Ho observed heterozygosity, He expected heterozygosity, PIC polymorphic information content,Nei: Nei's gene diversity index; I shannon wiener index, HWE deviations from Hardy–Weinberg equilibrium.

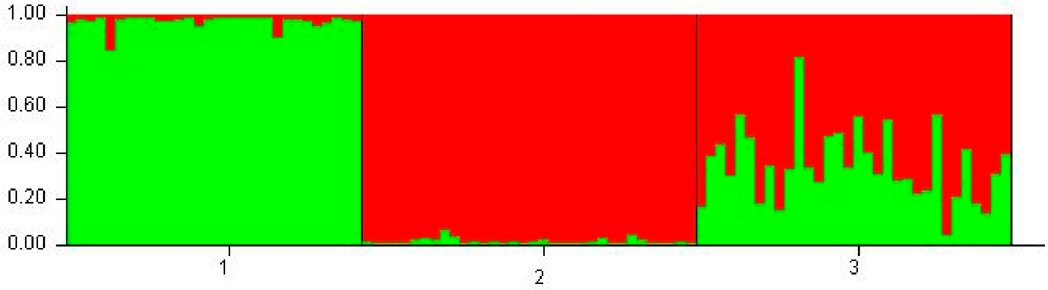

Figure S1. Genetic structure analysis of individuals from three populations under K=2 hypothesis. Each vertical bar represents an individual, with different colors indicating the proportional contribution of each K genetic cluster to the individual's genotype: 1. SM population, 2. SF population, 3. FM population.
